# Understanding students’ self-efficacy and motivation in sequential OSCE: a qualitative study

**DOI:** 10.1186/s12909-025-08301-5

**Published:** 2025-12-29

**Authors:** Jun Jie Lim, Chris Roberts, Shareen Nisha Jauhar Ali, Paul Collini, Amir Burney, Dyfrig Hughes

**Affiliations:** Division of Clinical Medicine, School of Medicine and Population Health, The University of Sheffield, Sheffield, UK

**Keywords:** Objective structured clinical examinations (OSCE), Sequential testing, Learner motivation, Self-efficacy, Medical Education, Clinical assessment, Medical licensing examination, Attribution theory, Student well-being

## Abstract

**Background:**

With the introduction of the UK Medical Licensing Assessment (UKMLA), many medical schools have adopted a sequential Objective Structured Clinical Examination (OSCE) format to evaluate clinical competence more efficiently. Sequential testing was designed to improve diagnostic accuracy and maximise the effectiveness of testing resources by administering a relatively shorter screening test for all students, followed by a confirmation test only for students who did not meet the passing threshold of the first test. While its psychometric robustness is well established, little is known about how students experience and interpret this assessment format. This study explores how psychological and contextual factors shape students’ self-efficacy, motivation and engagement within sequential OSCEs.

**Methods:**

Semi-structured interviews with 22 medical students were conducted following two full sequences of sequential OSCEs in the third and final year of study. Data were analysed using framework analysis informed by self-efficacy and attribution theory.

**Results:**

Students described multiple positive sources of self-efficacy, including sequential practice opportunities, peer and senior modelling, verbal reassurance, effective faculty-student communication and well-timed testing intervals. Negative influences included uncertainty about the format, emotional distress, unclear pass threshold, lack of assessment clarity, delayed feedback and insufficient support. Students who viewed sequential testing as a developmental opportunity reported higher confidence and adaptive attributions, whereas those who perceived it as punitive described heightened anxiety and reduced motivation. These patterns reveal an *anxiety–achievement paradox* — high objective success rates coexisting with persistent psychological stress.

**Conclusions:**

Sequential OSCEs can support learning when implemented with transparent communication, equitable access to preparation resources, and timely, supportive feedback. Attending to the emotional and motivational dimensions of assessment may improve the educational value of sequential testing and promote student well-being within high-stakes clinical assessment systems.

**Supplementary Information:**

The online version contains supplementary material available at 10.1186/s12909-025-08301-5.

## Introduction

National licensing examinations are a high-stakes hurdle for aspiring healthcare professionals [1, 2]. There is considerable variation in how these exams are conducted and the evidence supporting their effectiveness [3, 4]. A significant issue facing medical education is designing these assessments to be fair, reliable, and effective learning tools that accurately measure competencies without causing undue stress [5]. With the recent implementation of the national UK Medical Licensing Assessment (UKMLA) by the General Medical Council (GMC), which includes a high-stakes clinical examination similar to the United States Medical Licensing Examination, the focus on assessment methods in the UK is intensifying [6]. The UKMLA’s Clinical and Professional Skills Assessment is conducted in the penultimate or final year of the medical degree programme, and many institutions conduct a sequential objective structured clinical examination (OSCE) format for this purpose. Sequential testing was designed to improve diagnostic accuracy and maximise the effectiveness of testing resources by administering a relatively shorter screening test (i.e., the first part of the sequence) for all students, followed by a confirmation test (i.e., the second part of the sequence) only for students who did not meet the passing threshold of the first test [7].

Many previous studies have proven sequential testing to be reliable, robust and cost-effective [8–13]. However, little attention is paid to stakeholders’ perceptions and experiences. Understanding assessment perceptions is essential because it shapes motivation, learning approaches, and trust in the fairness and validity of exams. When assessments are perceived as unclear or inequitable, anxiety and disengagement may undermine performance and reduce educational value [5]. Smee and colleagues were the first to reveal negative perceptions of stakeholders in the administration of sequential OSCE in a Canadian licensure examination context [14]. Similarly, Duncumb and Cleland revealed students’ negative attitudes towards sequential OSCE at a Scottish University [15]. Although insightful, the limited studies have been primarily quantitative and cross-sectional, thereby lacking in-depth perceptions longitudinally within and throughout the sequential testing environment [16]. This has resulted in concerns and negative perceptions of sequential testing, such as heightened stress levels, anxiety, and perception of unfairness being repeatedly reported but not thoroughly investigated, particularly from a motivational and psychological perspective [17].

### Self-efficacy and attribution theory

The conceptual framework for this study is grounded in two well-established psychological theories: Bandura’s self-efficacy theory [18] and Weiner’s attribution theory [19]. Self-efficacy refers to individuals’ beliefs in their ability to successfully execute specific tasks, while attribution theory pertains to how individuals explain their successes or failures. A wealth of research reports the relationship between self-efficacy and student outcomes in health professions education. Self-efficacy is positively correlated with students’ mastery goals, motivation, self-regulated learning, and interprofessional collaborative performance [20–23], while negatively correlated with course-related anxiety, stress, and academic burnout [24–26]. Similarly, attribution theory has been widely used to explain students’ success or failure in high-stakes assessment settings [27, 28]. Despite their extensive use, no studies have systematically applied both self-efficacy and attribution theory within the context of sequential assessment.

In this study, self-efficacy and attribution theory serve as structured analytical frameworks for understanding students’ perceptions of their experiences in sequential OSCE. Following Cook and Artino’s summary of self-efficacy theory in medical education [29], we used the four major sources of self-efficacy—mastery experiences, vicarious experiences (senior modelling), verbal reassurance, and emotional states—to explore how students’ described their confidence and engagement in sequential testing. Weiner’s attributional dimensions—locus, stability, and controllability—were applied to interpret how students explained their performances and outcomes. By structuring our analysis through these theoretical categories, we identified patterns in how students’ self-efficacy beliefs and attributional interpretations intersected to shape their perceptions and engagement within the sequential OSCE.

### Study aim and research question

This study aims to explore OSCE students’ perceptions and experiences across the full testing sequence within a pilot national licensing examination setting, to understand how psychological and contextual factors shape engagement in high-stakes assessment environments.

Our specific research questions are:

- RQ1. What factors do students perceive as influencing their self-efficacy in a sequential OSCE?
- RQ2. How do students perceive their self-efficacy beliefs as shaping their engagement in a sequential OSCE?

These questions are important because they highlight the role of self-efficacy in shaping students’ motivation, confidence, and engagement within a high-performing assessment process. While sequential OSCEs are designed to enhance efficiency and reliability, understanding students’ perceptions can help optimise this effective system by addressing the psychological and communicative factors that influence how fairness and confidence are experienced.

## Methods

### Study design

This study adopts a qualitative descriptive design grounded in a post-positivist paradigm, which acknowledges that reality can only be approximated and that systematic, reflective inquiry enhances the rigour and credibility of qualitative interpretation [30, 31]. Guided by this paradigm, we employed a theory-informed framework analysis approach to systematically identify recurring patterns in students’ accounts of sequential OSCEs and interpret these in relation to self-efficacy and attribution theories. High self-efficacy was inferred when participants attributed success to internal, controllable factors, reflecting confidence and proactive engagement, whereas low self-efficacy was inferred when challenges were attributed to external, uncontrollable factors, indicating reduced confidence.

### Contextual background

The sequential OSCE is a large-scale, high-stakes exam used to determine pre-final year students’ progression through medical school and, for final-year students, their eligibility to graduate and obtain a provisional license to practice. The OSCE curriculum is based on the UKMLA content map, which determines “*the core knowledge*,* skills and behaviours needed for UK practice*”. The sequential OSCE is conducted annually at a purpose-built facility within a large sports hall for third and final-year medical students at two distinct time points. In our implementation of a two-stage approach, all students initially completed 12 stations (n1 = 636), with those scoring less than 2 x SEM above the borderline regression pass mark and failing to pass at least 8 out of 12 stations (n2 = 22) proceeding to an additional set of 12 stations for the confirmation sequence. The standard setting and decision-making process is modelled on the sequential testing framework developed by Pell et al. [13]. Students required to sit for confirmation will receive an email a week after the screening OSCE. The timescale between screening and confirmation is two weeks for third-year students and four weeks for final-year students. To account for the smaller numbers and doubling of test items, derivation for the cut score for the confirmation sequence is based on the reliability, standard deviation, and SEM of the screening sequence. The final pass mark for the combined OSCE is calculated as the average of the cut scores from both the screening and confirmation OSCEs, plus the SEM for the full examination. Students who failed the sequential OSCE overall would repeat the year, affecting approximately 0.5% of the cohort. To ensure consistency and fairness across all test items, the stations were blueprinted simultaneously with the main blueprinting process to assure equivalence. The stations assess a combination of clinical and communication skills over eight minutes for third-year students and ten minutes for final-year students. Examiners attended online or face-to-face training before the on-the-day briefing. Internal quality assurance examiners collaborated with external examiners throughout the assessment to ensure station performance and validity. Examiners with various levels of experience, from recent medical graduates to consultants, assessed two whole sequences across both year groups and were required to score student performance on the station-specific marking checklist, with a total of 25 marks. Simulated patients received training as part of a systematic Patients as Educators Programme. Students were briefed on the nature of sequential testing and how it works in principle and practice with lectures and written materials on the medical school learning management system.

### Data collection

We adopted semi-structured interviews to identify patterns and recurring themes across participants. JL and CR designed the interview schedule based on existing self-efficacy and attribution theories to ensure grounding in previously published literature and alignment with existing theoretical constructs (see supplementary material). To collect data representative of students’ experiences across different stages of their medical education, we conducted interviews with both third-year and final-year students who participated in the OSCE. We sent email invitations to all students at the conclusion of each sequence. Those who expressed interest were provided with a participant information sheet and gave written, voluntary consent before the interviews. Sampling was guided by the concept of information power, which considers the study aim, sample specificity, quality of dialogue, and analytic strategy when determining sample adequacy [32]. We initially aimed to recruit approximately 20–25 participants across the two student cohorts to ensure diversity while maintaining analytical depth. Recruitment and analysis proceeded concurrently, and data collection ceased after 22 interviews, when emerging data no longer contributed new insights relevant to the study aim, indicating sufficient information power had been achieved.

### Data analysis

We employed framework analysis, combining deductive and inductive approaches to improve rigour and accommodate the iterative nature of qualitative inquiry [33]. This method involves familiarisation, identifying a thematic framework, indexing, charting, mapping, and interpretation, enabling us to integrate predefined theoretical constructs while remaining open to emergent insights [34]. All interviews were transcribed verbatim by JL and SN, who re-listened to the recordings, re-read transcripts multiple times, and maintained reflective notes to record early impressions and assumptions. These notes were referred to throughout the analytic process to remain attentive to how the researchers’ perspectives might influence interpretation.

We employed multiple forms of triangulation to enhance credibility [35]. Investigator triangulation involved JL and SN independently coding transcripts, then engaging in weekly reflexive meetings to discuss coding divergence collaboratively. During meetings, both researchers articulate their interpretive reasoning and link it to the theoretical framework and contextual meaning of participants’ accounts. Consensus was reached through iterative discussion and reflection, prioritising theoretical coherence and representativeness of the data. This approach aligns with qualitative guidance that cautions against treating inter-coder reliability as a proxy for rigour and instead emphasises negotiated meaning through reflexive discussion [36].

This early phase was deliberately inductive to remain open to unexpected insights, acknowledging that post-positivism values both theory-driven inquiry and the iterative exploration of emergent patterns to refine or challenge existing frameworks. Data were managed using NVivo (QSR International V14, 2023) to support systematic coding. Following this inductive phase, axial coding was applied to identify relationships between codes, and to develop broader categories capturing key patterns in students’ experiences. The analysis then became more deductive. We drew inferences based on the language and behaviours described by participants in their narratives. For example, when students spoke about overcoming challenges or successful performances, we categorised these as indicative of high self-efficacy. Similarly, expressions of doubt or fear were interpreted as lower self-efficacy beliefs. Attribution theory was also applied in a structured manner, using Weiner’s dimensions of locus, stability, and controllability to categorise how students explained their success or failure in the OSCE [19]. We also employed theory triangulation by integrating insights from both self-efficacy and attribution theory, using each framework to interpret different dimensions of students’ responses to the OSCE experience.

To further enhance rigour, three researchers (JL, SN, and CR) independently re-analysed portions of the data to refine and develop a final analytical framework. JL and SN then applied this structure to the remaining transcripts, while maintaining reflexive discussions to ensure interpretations remained grounded in the data. Data triangulation was also achieved by comparing third- and final-year students’ perspectives to capture how beliefs evolved across stages of medical training. Throughout the analysis, we remained attentive to alternative explanations and instances where participants’ narratives did not fully align with dominant patterns of self-efficacy and attribution, acknowledging the potential for data to refine or challenge existing theories. CR provided methodological oversight and guided the application of self-efficacy and attribution theory within the broader contextual analysis. This recursive movement between data and theory is consistent with the post-positivist emphasis on refining existing knowledge while remaining open to alternative explanations. The combined use of investigator, theory, and data triangulation thus represents a rigorous, structured approach that strengthens the credibility and trustworthiness of our findings.

### Reflexivity

The research team comprised six members with diverse educational, clinical, and research backgrounds. Two members (JL and SN) were recently graduated foundation doctors with first-hand experience in sequential testing, offering valuable insider perspectives on the assessment process. Three members (PC, AB and DH) were clinicians with extensive experience in implementing and evaluating large-scale OSCEs, while CR, a professor of medical education, provided methodological oversight and expertise in qualitative and assessment research.

We recognised that these varied perspectives could shape our interpretations. Insider experience may have heightened sensitivity to stress and fairness concerns, while examiner and assessment roles might predispose researchers to defend assessment practices. Likewise, three authors trained in the Global South (JL, SN, and AB) brought an awareness of equity and access issues that influenced our focus on systemic factors. To address these potential biases, we maintained reflexive notes, engaged in regular team discussions to challenge assumptions, and iteratively reflected on how our positions shaped interpretation. This process of collective reflexivity supported balanced analysis and enhanced the credibility of findings [37].

### Ethical approval

This study received approval from the University of Sheffield Medical School Ethics Committee (Reference number: 057035). All participants received written information about the study and provided informed consent for their anonymised responses to be used in publication. Participation was entirely voluntary, and students were assured that their decision to participate or withdraw would not affect their academic standing. To minimise potential risks and discomfort, participants were informed that they could skip any questions or end the interview at any time, and were provided with contact details for the student representative for contact should they experience stress related to the OSCE. To reduce potential power dynamics, all interviews were conducted by JL, an academic foundation trainee, who was not involved in teaching, assessment, or grading of participants. This approach helped ensure that students could speak freely and without concern for academic consequences.

## Results

### Participant characteristics

Twenty-two semi-structured interviews were held between November 2023 and May 2024, spanning the duration of two complete sequences of testing. The students interviewed included those who only sat for the first sequence and those who sat for both sequences. Participant demographics are presented in Table 1 to provide contextual understanding of the sample.

**Table 1:** Participant demographics

	Age group		Gender		Ethnicity			Total
	18–24	25–34	Female	Male	White	Asian or Asian British	Black British, Caribbean or African	
Year 3	9	1	9	1	4	5	1	10
Year 5	11	1	8	4	9	2	1	12

### RQ1 what factors influence students’ self-efficacy in a sequential OSCE?

In answering our first question, we constructed our subthemes and organised them into two categories: self-efficacy sources and contextual task-engagement factors, which collectively mediated students’ pre-sequence self-efficacy beliefs. We tabulated illustrative quotes that support the analysis below.

### Self-efficacy sources of influence

Students’ self-efficacy beliefs were categorised into four primary sources of influence: sequential practices, vicarious experiences, verbal reassurance and emotional states. Students found that sequential practices increased their self-efficacy: “having those practice runs helps me feel more confident”. However, several students raised socioeconomic equity concerns, noting that access to paid mock OSCEs created disparities in confidence: “if you had more money, you were more likely to get onto a mock compared to someone else” (see Table 2, quotes 1 and 2). This suggests that unequal access to preparatory opportunities may shape the development of self-efficacy and contribute to perceived inequities in assessment readiness.

**Table 2:** Illustrative quotations for students’ self-efficacy sources in sequential testing

1	I think that I’ll probably pass the screening [first part of the sequence] because besides placements, I already had three more mock sequential OSCEs under my belt and I scored about 80% in all of them. I think definitely having those practice runs help me feel more confident and makes me feel like I should not have to sit for the confirmation [second part of the sequence]. (Student 14, female, year 5)
2	It was just the fear of unknown that I was stressing over what’s going to happen, and how is it going to be, and I think having a mock [practice sequential OSCE] is probably the most helpful that I’ve found in terms of what the structure will be and what to expect, but even then the amount you can do is limited because obviously they’re run by societies, and they charge, so it’s like I pay 10 pounds each time, and then **it just felt like**,** if you had more money you were more likely to get onto a mock compared to someone else**. Do I think it’s unfair? 100%! Because I did two mocks, I wanted to do more, but I can’t afford to keep paying. (Student 6, female, year 3)
3	We had a really good teaching where an F1 (foundation year 1 trainee doctor) basically explained her process and I think actually just **hearing it from someone that has done it** was really helpful because I feel like she definitely understood the questions that people had a lot more than the doctor that gave us a talk about the OSCE. That session definitely made me feel a lot more assured. (Student 16, female, year 5)
4	The medical school **stresses every year how many people pass the first one**, and how little people have to do confirmation, I’m **really grateful that they stress to us** because that was really comforting and like when I came out, it was what I kept thinking about was like “oh, so many people pass like, no one really fails. (Candidate 4, female, year 3)
5	The medical school is really good in how they went about it, cause the meeting with med school after my friend didn’t pass the screening OSCE was like **you didn’t fail**, we just want to see more of you, you were so close to pass, we just want to make sure you’ve got enough, it is a lot more of a like **you’re good**,** we just want to make sure you’re great**. (Student 7, female, year 3)
6	I think sequential does kind of give you a sense of relief and I would say on a whole lot less stress because if we failed, we could have that second sitting where we could just repeat it, and nothing terrible would happen. and it wouldn’t get marked down. So I think a couple of people I talked to were like, ‘Oh, yeah, it’s fine, like, even **if it doesn’t go the way I wanted to do**,** I’ll just do the second sitting**’. (Student 6, female, year 3)
7	The sequential format is new, we didn’t know about it before, I think because from A-levels [school-leaving qualification], you’re always used to resits as an idea. So, I think it’s very hard to get that concept out of your head. I think it’s tricky because I think **people will always see it as a sort of resit**, essentially they are doing the exam again, because they haven’t done well enough in the last one. I guess we need more information on it, like simple information explaining why they’re doing it so people can see the reasons for it, why the Med school has chosen to do it. (Student 11, female, year 5)
8	I think the fear is that what if I pass the OSCE, but I didn’t pass it enough and have to sit for confirmation, then what if I passed confirmation, but then didn’t pass enough again, so **I had two passes**,** but they weren’t above the confidence interval**,** then that would be a straight fail** which I think is difficult to wrap my head around. (Student 16, female, year 5)

Students also turned to their seniors for support, finding value in “hearing it from someone that has done it”, which increased their confidence (see Table 2, quote 3). Verbal persuasion and reassurance from the medical school played a crucial role in fostering self-efficacy. Positive messaging, such as framing borderline performance as a growth opportunity, made students feel like “you didn’t fail… we just want to make sure you’re great” (see Table 2, quote 4 & 5). Sequential testing was reported to alleviate anxiety, with students explaining that “if it doesn’t go the way I wanted, I’ll just do the second sitting (see Table 2, quote 6). However, some students experienced a lack of familiarity with the sequential format, noting that “people will always see it as a sort of resit”, which undermined their confidence (see Table 2, quote 7). Finally, concerns over unclear passing thresholds added to students’ anxiety. As one student described, “I had two passes, but they weren’t above the confidence interval, then that would be a straight fail” (see Table 2, quote 8).

### Contextual task-engagement factors

Alongside self-efficacy sources, contextual task-engagement factors collectively influence students’ engagement with the testing process and their self-efficacy, thereby impacting their motivation and performance. Effective communication about sequential procedures was cited as an important factor in boosting students’ self-efficacy. One student expressed how they felt well-prepared because “we all went into the examination period knowing what was happening” (see Table 3, quote 1). Timely sequencing by reducing the time gap between sequences also helped some students maintain momentum, with one commenting, “I wasn’t relaxed, so I was able to just jump back into revision” (see Table 3, quote 2).

**Table 3:** Illustrative quotations for students’ task engagement factors in sequential testing

1	We were given the opportunity to ask questions about sequential. I’ve attended probably four lectures that included Q&A sessions about sequential. There was also the podcast that talks about it, which I thought was quite useful. I think the medical school did as well as you could expect for them to communicate what this was so I feel like we all went into the examination period knowing what was happening and what the system would be. (Candidate 13, male, year 5)
2	I thought that since there was only one week gap between sequences to prepare and I was in exam mode, **I wasn’t relaxed**,** so I was able to just jump back into revision**, but if I had a month off, I probably would have gone very relaxed and probably gotten more stressed in that one month’s time. (Candidate 9, male, year 3)
3	I think everyone’s probability of passing is slightly lower in sequential because to pass you have to **get above two confidence intervals above the pass mark** and I think that puts more **pressure on everybody** because that’s a big percentage. (Candidate 11, female, year 3)
4	I don’t know why they [medical school] introduced a sequential OSCE …… I still **don’t really understand the difference between screening and confirmation**, and like comparing it to first sitting and resit, I don’t really see how it’s different. (Candidate 4, female, year 3)
5	What I didn’t like about the sequential was the **lack of understanding** that going into it, none of us knew what the pass mark was and exactly how the first OSCE worked so we didn’t quite know how to pass the first time around and not have to do the second OSCE. And so **there were a lot of unknowns for us**. So, it felt like the med school had organised a sequential OSCE for us but not actually told us how to navigate it. (Candidate 21, male, year 5)
6	I think the bits that I was unsure about sequential, I’ve kind of **gone digging for a bit more information**. I think knowing that information made me feel more confident where my peers didn’t know where to find that information, and so we’re **getting quite worried about how the whole thing works as a structure** and……I think that **component of stress was quite high**. (Candidate 14, female, year 5)
7	I just think that a good clarification of how sequential would be beneficial for us, because I don’t understand how the math works out that more people will pass. It would have been nice to know what the numbers actually are so we would be able to fully appreciate the system and understand the benefits. Because **without knowing those numbers**,** the benefits are meaningless**. (Candidate 21, male, year 5)
8	Being given the news that I’d had to sit the second sitting would really **throw me off** because I don’t have that feedback. I don’t know what I did wrong. I’d be really keen to know what I’d done wrong. That would be like the main thing that I’d really need. I’d **really want feedback** before I then went into the next sequence. (Candidate 4, female, year 3)
9	My friend **found it so stressful because she didn’t know quite how badly she’d done** in the first part of the sequence, whether she failed outright completely or whether she’d only just sort of passed but not quite gotten above the standard deviations, she didn’t know whether she needed to completely redo her revision or whether to just do a little bit on top of what she would normally do. (Candidate 22, female, year 5)
10	I don’t actually know what happens for the confirmation. Does someone then get in touch? Or how does it run? **I don’t know if there’s any extra support at all**. It felt like we were just on the edge of trying to pass the first time so that we didn’t have to think about it. (Candidate 16, female, year 5)

However, several negative factors hindered students’ task engagement. Sequential testing was perceived as more challenging due to the higher pass mark required in the first sequence, leading to “pressure” among students (see Table 3, quote 3). Many described a lack of transparency and communication regarding the rationale and calculation for this threshold, which made the benchmark feel arbitrary and unattainable. This uncertainty undermined confidence and contributed to perceptions of unfairness. Additionally, many students struggled with a “lack of understanding” regarding the mechanics of sequential testing and pass mark setting (see Table 3, quotes 4 & 5), with one student citing, “we’re getting quite worried about how the whole thing works as a structure” and felt they had to “go digging for a bit more information” to reduce stress (see Table 3, quotes 6). Some candidates expressed a need for clearer explanations about the benefits of the sequential testing format. One student pointed out that “without knowing those numbers, the benefits are meaningless” (see Table 3, quote 7). Students commonly indicated that communication should include clearer explanations of how the pass mark is determined, what constitutes borderline performance, and how the second sequence is intended to function as a developmental opportunity rather than a punitive resit.

Feedback was another critical factor influencing task engagement and self-efficacy. Several students felt that withholding feedback between sequences was anxiety-inducing. One student shared, “I’d really want feedback before I then went into the next sequence” (see Table 3, quote 8). The absence of clear feedback left some students unsure about how to approach their preparation for the second sequence, with one expressing, “she didn’t know whether to completely redo her revision or just do a little bit more” (see Table 3, quote 9). Finally, uncertainty about available support further diminished self-efficacy for students needing to sit for the second sequence. “I don’t actually know what happens for the confirmation… Is there any extra support at all?” one student asked, reflecting the lack of clarity on what resources might be available (see Table 3, quote 10).

### RQ2 how do students perceive their self-efficacy beliefs as shaping their engagement in a sequential OSCE?

Students described ways in which their perceived self-efficacy related to their motivation and engagement in the sequential OSCE, as reflected through efficacy-activated and attributional patterns in their narratives. We observed patterns in students’ narratives that reflect behaviours or attitudes associated with varying levels of self-efficacy. Importantly, students’ self-efficacy was not static, and these fluctuations were influenced by various contextual factors and emotional responses before, during, and after the assessment.

### Efficacy-activated processes

Efficacy-activated processes refer to the mechanisms by which an individual’s self-efficacy beliefs influence their perceptions, behaviours, and emotional responses within a sequential testing framework. These processes illustrate how students perceived their self-efficacy as influencing how they interpreted and reacted to different aspects of the testing experience, shaping their reported motivation and emotional responses.

In terms of cognitive processes, students who displayed confidence in their abilities often saw sequential testing as a “safety net” that reduced pressure, providing “two chances” rather than a single high-stakes event (see Table 4, quote 1). This perception allowed them to view the second sequence as an opportunity for improvement, aligning with higher self-efficacy patterns. Conversely, students who interpreted the second sequence as a “resit” and an indicator of failure tended to experience lower confidence, with one student noting that this mindset “feeds fear and stress” (see Table 4, quote 2). These contrasting interpretations illustrate perceived differences in how students with varying levels of self-efficacy appraised the sequential format.

**Table 4:** Illustrative quotations for candidates’ efficacy-activated processes in sequential testing

1	I think it is really helpful to have the sequential format because it took a lot of pressure off, I know you had to do better than normal, but it felt like a screening, a first try, a safety net, ‘I don’t do well, that’s fine. I just have to sit more exams’ and it is not a fail, rather than a complete ‘okay, you have to pass this’. You get two chances, it doesn’t make you doubt yourself and your capability, it also takes off the distress and really helped me and all my friends. (Candidate 19, female, year 5)
2	Sequential testing definitely **felt like a resit**, it was just the **same thing repeated** as opposed to a continuation of the exam, so I revised for confirmation as if I had failed anyways and failing it’s a very big thing as a student mentally, it is something that **feeds fear and stress**, and makes you forget and not very prepared. (Candidate 9, male, year 3)
3	My friends who have to sit for confirmation were very sort of hunkered down and kind of practiced on their own really and prior to the confirmation, I think everyone that I knew that was doing the confirmation OSCE was **feeling very low and kind of just stuck to themselves** although we did all offer to help but I think they just found it so stressful. (Candidate 22, female, year 5)
4	Certainly I would **prefer sequential versus doing two days’ worth of OSCE**, I was quite confident that I would pass the first time round and talking to people I know … granted that they passed at screening, I’d be surprised if people prefer the alternative with more stations. (Candidate 21, Male, year 5)
5	It **feels like there’s a higher line to hit**, you can’t just pass, you need to pass it by a substantial amount. I’ve **felt a bit more uncertain weirdly**,** it’s kind of terrifying**, I’d rather have pass-fail where I just know I have to resit than a second sequence to confirm my abilities. It feels a bit scarier. (Candidate 22, female, year 5)
6	I think I would rather have more stations and not have to be so much above the pass mark. I think it would be more helpful to do more stations just to see more different scenarios because then each of them isn’t worth so much, and then know that it was a lower criteria to pass, because in sequential you **couldn’t afford to mess up even a bit**. (Candidate 11, female, year 3)

Affective processes also played a key role. Students who expressed positive emotions, such as relief or enthusiasm, seemed better able to cope with the isolation of preparing for the second sequence. In contrast, those who experienced “feeling very low” or anxious often had lower self-efficacy, finding the preparation process particularly stressful (see Table 4, quote 3). This pattern suggests that students who perceived themselves as having lower self-efficacy also described more anxiety and stress during the preparation phase.

In terms of selection, students who displayed higher confidence in their abilities often reported a preference for sequential testing, suggesting that they viewed the first sequence as an attainable challenge (see Table 4, quote 4). Conversely, students with lower confidence preferred the traditional pass-fail structure, feeling that sequential testing imposed a “higher line to hit” and created more uncertainty (see Table 4, quote 5). Another student mentioned that the higher pass mark meant “you couldn’t afford to mess up even a bit” (see Table 4, quote 6). These patterns highlight associations between differing self-efficacy beliefs and students’ preferences for testing formats.

### Attributional processes

Attributional processes refer to how students interpret their performance and assign causes to their outcomes during and after the OSCE. Students who attributed their performance to controllable internal factors, such as personal preparation, often described behaviours aligned with higher self-efficacy (see Table 5, quote 1). This reflects the sense of control and personal responsibility typical of students with high self-efficacy.

**Table 5:** Illustrative quotations for candidates’ attributional beliefs in sequential testing

1	I guess how much I knew my content, and I suppose, like my own knowledge, that was the biggest thing that affected my performance because obviously, subjectivity is like, as I said, it’s literally everywhere, every person faces subjectivity in the OSCE, so it might at some point cancel out, but the most important thing that’s like floating everybody in the game is, how much you’ve prepared, how much you revised, how well do you know your content? It’s just my own thing, really … (Candidate 1, female, year 3)
2	OSCE is very subjective. I spoke to my friend, and there were so many differences in our experiences at the same station. It just doesn’t feel equal across the board; for everyone, it’s’ very actor-dependent. I think **the subjectivity is just unfair and causes stress**, it obviously affects my personal satisfaction, because it makes me feel like I probably haven’t studied hard enough, and that’s why I probably didn’t get the grade I wanted. (Candidate 7, female, year 3)
3	I felt like the biggest pitfall in the first sequence is just the fact that **every circuit was so different**. My worry was I am going to end up in the second sequence of testing just because I was given a tweaked scenario with different standards than someone else. I definitely felt that when I came out. It was the same station, but the actors had given different information. So yeah, I just got the worry that if something like that happens again on the next part of the sequence, it’s out of my control. (Candidate 16, female, year 5)
4	I remember coming out and being like ‘this is why they’ve got a second sequence’, because I think you could really mess up just because of the **nerve-racking and chaotic atmosphere** … I tried to be empathetic with patients, but I felt the patients weren’t really responsive like in a real environment; I couldn’t really develop that rapport with them; I just felt everything I was doing was really strange. (Candidate 2, female, year 3)
5	For someone like me, I struggle with these exams because of mental health, so I think I probably fit that category of people who would probably pass but don’t pass enough and have to sit for confirmation. It felt difficult to be told that you could do enough, but it still wouldn’t be enough to pass in the first part of the sequence. I do struggle with that (Candidate 16, female, year 5)
6	During the confirmation, there’s less students so it’s a little quieter, more relaxed, and the exam ran a lot more smoothly as well, so I wasn’t as nervous. Also, the patients were a lot more engaged in my opinion. (Candidate 9, male, year 3)
7	I would say the confirmation was quite well structured or organised, there weren’t any sort of logistical issues or things running over time, which would have added extra stress on the day, **everything went smoothly**. (Candidate 20, male, year 5)
8	I was being very slow in my examination, but the examiner was trying to hurry me up; it is good he did that because that’s the only time I finished, whereas the other times, I wouldn’t be able to. (Candidate 14, female, year 5)
9	Actually, in confirmation, the examiners did give me more opportunities to answer questions. If I got something wrong, they would let me have **multiple attempts at answering**. It was nicer in that regard. (Candidate 9, male, year 3)

In contrast, students who attributed their performance to external, uncontrollable factors, such as variability in the testing environment or inconsistencies in examiner behaviour, expressed frustration and stress, which aligns with lower self-efficacy patterns. For example, students shared concerns about the variability in scenarios, noting that “every circuit was so different”, describing how differences in actor performance and station conditions “don’t feel equal across the board” (see Table 5, quotes 2, 3 & 4). These quotes illustrate how external attributions contribute to feelings of helplessness and lower self-efficacy. Students who made self-effacing attributions, such as attributing failure to stable internal factors like mental health, often experienced a further decrease in confidence and motivation for future tasks. One student mentioned that they “struggled with these exams because of mental health” (see Table 5, quote 5). This highlights how personal factors, combined with a perceived lack of control, diminish self-efficacy.

Some students described the environment in the second part of the sequence as more conducive to better performance. Improvements in organisational factors and logistical efficiency during the confirmation sequence reduced stress and allowed them to perform more confidently (See Table 5, quotes 6 & 7). Furthermore, supportive examiner behaviour, such as being given “multiple attempts at answering” questions, helped students build their confidence during the confirmation sequence (see Table 5, quotes 8 & 9).

## Discussion

### Summary of key findings and comparison with existing literature

Our study provides a comprehensive exploration of students’ perceptions of sequential testing through the lens of self-efficacy and attribution theory. Despite the assessments’ high pass rate, students reported persistent anxiety and uncertainty – a tension we describe as the anxiety–achievement paradox in sequential assessment. This paradox highlights how psychological responses may diverge from objective outcomes in high-stakes contexts [38]. Our findings extend previous psychometric research [7–10, 39] by introducing a theoretically grounded understanding of how psychological and contextual factors shape students’ experiences of sequential OSCEs. Self-efficacy was influenced by both personal and contextual elements, such as familiarity with assessment formats, senior modelling, and communication clarity, which aligns with Bandura’s concept of reciprocal determinism [18]. Students who perceived sequential testing as an opportunity for improvement displayed higher self-efficacy and internal, controllable attributions, whereas those expressing doubt attributed difficulties to external, uncontrollable factors, consistent with Weiner’s attributional dimensions [19]. This reciprocal relationship between attribution and efficacy highlights the dynamic and context-specific nature of motivation in high-stakes assessments [40, 41].

**Fig. 1:**
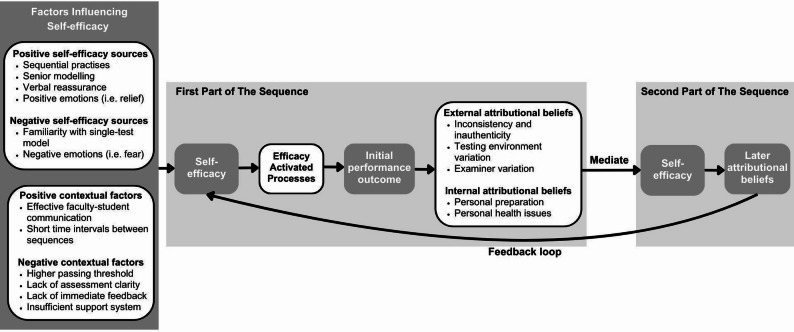
Student’s self-efficacy and attributional beliefs in a sequential OSCE setting. Adapted from Cook and Artino [29]

Drawing on these findings, we developed an adapted socio-cognitive framework of motivated learning (Fig. 1), integrating self-efficacy sources, contextual factors, and attributional processes identified in this study. The model illustrates the reciprocal relationship between self-efficacy and attribution across sequential OSCE stages, offering practical and theoretical guidance for developing strategies to enhance students’ well-being and performance in sequential testing contexts.

Our study extends the literature on attributional processes, showing how students primarily attribute unsatisfactory engagement to external factors related to OSCE and sequential testing, such as variability in the testing environment or examiner behaviours. We discuss socioeconomic equity concerns, emphasising that students’ perceptions of fairness were primarily linked to systemic and contextual factors, such as unequal access to preparatory resources rather than individual differences in self-efficacy [42]. This finding illustrates how unequal access to paid mock OSCEs reinforces disparities in self-efficacy and preparedness. Institutional measures, such as free or standardised preparatory workshops, could help ensure equitable opportunities for confidence-building. We conceptualise fairness as the provision of equitable assessment conditions that allow all students comparable opportunities to demonstrate competence. In this context, perceived unfairness arises when institutional practices, such as unclear communication, inconsistent feedback, or unequal resource access, shape students’ self-efficacy. Addressing these factors can improve both the perceived legitimacy and educational impact of high-stakes assessments.

While students frequently expressed concerns about transparency and uncertainty in sequential testing [15], it is important to distinguish between legitimate expectations for clear communication and the inherent ambiguity of complex assessment systems. Performance-based assessments inevitably involve contextual uncertainty, which should not be mistaken for unfairness [43]. Some of the anxiety reported by students may also reflect contemporary expectations for rapid feedback rather than deficiencies in the sequential testing format itself [44]. Our findings align with the Medical Council of Canada’s licensure experience, which underscores the importance of effective communication between faculty and students to ensure a shared understanding of the sequential process [14]. Our findings reinforce existing evidence that unclear communication and limited feedback can erode students’ self-efficacy [16]; thus, institutions should prioritise clarity around the purpose and structure of sequential testing. Although aligning communication with student preferences can enhance engagement, meeting all preferences may not necessarily improve psychometric quality [45]. Balancing transparency and fairness with the validity and integrity of assessment design remains a key challenge for educators and policymakers.

### Methodological strengths and limitations

Our study has several methodological strengths and limitations to consider. It is the first to explore students’ in-depth understanding of a sequential OSCE within a national licensing examination setting, contributing to the efficiency and effectiveness s of high-stakes licensure decisions. We adopted a theoretically grounded and systematic approach, which improved rigour in our data collection and analysis and the credibility of our findings. The detailed description of our research procedures further enhances the transparency and replicability of our work, and our strong conceptual framework and clearly articulated theoretical framework enable our work to be well-situated within the existing programme of research on sequential testing and allow readers to assess the applicability of our findings in other contexts. We collected the views and experiences of junior and senior clinical medical students over 22 interviews, representing, we believe, a sample with sufficient information power, considering our focused aims, tight sample specificity, rich interview dialogues and theoretically-driven framework analytical approach. Rigorous reflexive practices further demonstrate post-positivist rigour and help mitigate biases. Specifically, the research team sought to minimise insider bias (arising from two researchers’ prior experience as OSCE candidates), confirmation bias (by systematically challenging emerging interpretations through investigator triangulation), and interpretive bias (by documenting analytical decisions in audit trails and revisiting coding frameworks through team discussion). Additionally, the use of multiple forms of triangulation reflects the post-positivist emphasis on enhancing the confirmability of findings through systematic cross-checking and reflexive interpretation.

However, the reliance on qualitative narrative data introduces potential interpretive bias. Although we used multiple quotes and triangulated findings to increase robustness, future research could incorporate quantitative measures of self-efficacy to complement our findings. Our study focused on UK undergraduate medical OSCEs, which may limit the transferability of our findings to postgraduate or non-UK OSCE settings. Additionally, our sample was predominantly female, reflecting typical gender demographics in medical education, which may limit applicability to more gender-balanced or male-dominated cohorts. However, no meaningful differences in perceptions were observed between male and female participants in this study. Sampling bias is another limitation, as we only included students who passed the overall sequence, excluding those who failed. Future research should explore the perspectives of those who failed to provide a more comprehensive understanding of self-efficacy. Longitudinal studies could further illuminate how perceptions evolve with repeated exposure to sequential testing.

### Implications for future research

As Swanson and Roberts [1] noted, the prevalence of OSCE-type assessments in national licensure examinations is expected to increase. To ensure the adoption of best evidence-based practices internationally, researchers should consider combining both qualitative and quantitative approaches to examine key factors influencing student outcomes. Quantitative scales could be employed to statistically prove relationships, such as the impact of feedback mechanisms on student self-efficacy and attributional beliefs. These insights could inform the design of targeted interventions. Interventions aimed at enhancing student self-efficacy and internal attributions of success through structured feedback and preparatory activities could be developed and tested. Pilot programs could assess the effectiveness of such interventions in reducing stress and improving performance in high-stakes exams like OSCEs. Longitudinal studies would further contribute to understanding changes in students’ self-efficacy over time, particularly in relation to sequential OSCEs and long-term learning outcomes. Moreover, expanding research to include diverse student populations, such as nursing, physician associates and dentistry, and in varying educational contexts, especially in non-Western countries, is crucial. This expansion could help ascertain the transferability of findings across different educational systems. Additionally, exploring additional theoretical perspectives that can deepen understanding of how students perceive and respond to sequential testing formats could be valuable.

### Implications for educational practice

This study offers critical insights for educators and policymakers aiming to enhance the fairness and effectiveness of high-stakes sequential assessments internationally. Our findings emphasise the necessity of transparent communication regarding sequential testing to counteract stakeholders’ unfamiliarity with the format. In particular, students expressed a need for clearer communication about how passing thresholds are determined, the rationale for sequential design, and what progression to the second sequence signifies. Framing the confirmation sequence as a developmental opportunity rather than a punitive resit could help reduce anxiety and strengthen students’ self-efficacy.

Providing accessible preparatory opportunities, such as free or standardised mock OSCEs, could help address socioeconomic disparities in preparedness. Consistent with prior work [5], we also suggest that OSCE designers focus on enhancing the authenticity and standardisation of simulated environments to improve the conduct and perceived fairness of sequential testing. Finally, recognising the psychological impact of high-stakes assessments, institutions should strengthen student support structures, including mentorship, peer networks, and timely feedback, to promote confidence and well-being throughout the testing process.

## Conclusion

This study provides new insights into how medical students experience sequential OSCEs by examining the interplay between self-efficacy, attributional beliefs, and contextual factors within a high-performing assessment system. Despite the process’s psychometric robustness, students reported persistent anxiety and uncertainty – an anxiety–achievement paradox that highlights the psychological complexity of high-stakes assessment environments. By illustrating how communication, feedback, and resource access shape perceptions of fairness and confidence, our findings emphasise the importance of addressing the emotional and motivational dimensions of assessment to support student well-being in health professions education.

## Supplementary Information

Supplementary Material 1

## Data Availability

The datasets generated and/or analysed during the current study are not publicly available due to privacy and ethical concerns, but are available from the corresponding author on reasonable request.
