# Supplementary material for: Understanding students’ self-efficacy and motivation in sequential OSCE: a qualitative study
Source: BMC Med Educ. 2025 Dec 29;25:1736. doi: 10.1186/s12909-025-08301-5 (PMC12751480; doi:10.1186/s12909-025-08301-5)
Supplement: Supplementary file 1 — Supplementary Material 1 [file 12909_2025_8301_MOESM1_ESM.docx]

**Appendix S1: Final interview guide.**

1. **What are your thoughts about the sequential OSCE format**?
2. Can you describe the benefits of the sequential format?
3. Can you describe the issues and pitfalls of the sequential format?
4. Can you describe the difference between sequential and single-stage assessments?
5. Are there any specific aspects of the sequential testing format that may cause stress?
6. **What are your thoughts and experiences about the sequential OSCE?**
7. How did you think you performed?
8. Were there any unexpected consequences?
9. What do you think about the fairness of the assessment?
10. **How did you prepare for the sequential OSCE?**
11. How did you feel about preparing?
12. Any particular strategies that were helpful
13. **What do you think are the factors that affect your performance in the sequential OSCE?**
14. How well do you think you have done?
15. How do you come to this conclusion? What factors affected your judgement of how well you have done?
16. **How confident are you in passing the first part of the sequence?**
17. How do you make that judgement? What factors influence your confidence?
18. Would your likelihood of passing OSCE be affected by whether it is a sequential format or not? Why do you think so?
19. **What do you think of a confirmation circuit?**
20. How are you feeling about the possibility of having to take a confirmatory test?
21. How does that make you feel?
22. **Is there an alternative format you prefer for the OSCE other than a sequential format? Why? Or why not?**
23. **Is there anything else you would like to share regarding the sequential OSCE format?**
